# Teaching Neuroimages: Dynamic Digital Subtraction Myelography Discloses a Ventral CSF Leak in a Patient with Upper Limb Amyotrophy

**DOI:** 10.1007/s00062-022-01210-w

**Published:** 2022-09-05

**Authors:** Niklas Lützen, Anna Zeitlberger, Jürgen Beck, Horst Urbach

**Affiliations:** 1 Dept. of Neuroradiology, Medical Center. University of Freiburg, Breisacher Str. 64, 79106 Freiburg, Germany; 2 Dept. of Neurosurgery, Medical Center. University of Freiburg, Freiburg, Germany

In response to the article “Ventral Longitudinal Intraspinal Fluid Collection Presenting as Upper Limb Amyotrophy” [1] we would like to point out the importance of sophisticated examinations in order to find a spinal leak in spontaneous intracranial hypotension (SIH).

In 2018, Nicoletti et al. reported on a 63-year-old man with bibrachial amyotrophy due to a spinal cerebrospinal fluid (CSF) leak, which was not located at this time [1]. Dynamic digital subtraction myelography with the patient in a prone position now showed a ventral leak at Th 6/7.

**Fig. 1:**
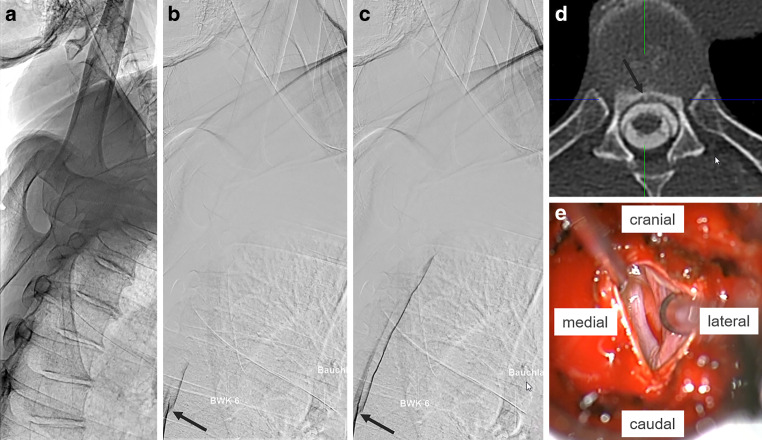
Native lateral X‑ray of patient in prone position with arms overhead in an angiographic suite and a combination of body and table tilting of 20° head down. Left shoulder (humeral head) is elevated over the level of the spinal canal (**a**). Digital subtraction myelography (*DSM*) in the same position. Incoming contrast agent enters the ventral epidural space at the level of tear at Th 6/7 (*arrow* in **b**). As the DSM progresses, contrast runs faster toward the head outside the intrathecal space than inside, indicating a large leak (**c**). Axial spinal CT post myelography with a small bony spur (*arrow*) at the level Th 6/7. The ventral epidural space is filled with contrast agent (**d**). Intraoperative view from dorsal with a large tear of about 5 mm in the ventral dura in craniocaudal orientation (**e**)

Dynamic digital subtraction myelography is the preferred imaging modality to locate a ventral CSF leak [2]. As most ventral leaks are located in the upper thoracic spine, positioning of the patient with elevation of the shoulder is important (shown in Fig. 1a–c).

Bibrachial amyotrophy is a very rare manifestation of spontaneous intracranial hypotension (SIH) and likely caused by stretching of the cervical nerve roots over the extradural CSF collection [3]. Large extradural CSF collections that may progress over the years suggest a large leak rendering the exact localization even more challenging. Minimally invasive surgery from the back of the spine revealed a distinct tear with an underlying small bony spur (shown in Fig. 1d, e).
